# Literary, Found and Research Poetry: New Approaches to Representations of Aging and Aged Care

**DOI:** 10.1093/geront/gnad056

**Published:** 2023-05-12

**Authors:** Sarah Holland-Batt, Evonne Miller

**Affiliations:** School of Creative Practice, Queensland University of Technology, Brisbane, Queensland, Australia; School of Design, Queensland University of Technology, Brisbane, Queensland, Australia

**Keywords:** Agism, Arts, Creativity, Nursing homes, Related therapy

## Abstract

At a time when rapid population aging is producing an emphasis on questions of healthy aging in the public discourse, conditions such as dementia, physical, and other disabilities still too often remain taboo, and this is particularly true in relation to the confronting subjects of aged care, neglect, and failures of care provision. This article considers the transformative potential of 2 different but complementary forms of poetry—research poetry and lyric poetry—to break these silences and represent experiences across the physical and emotional spectrum of aging, including the perspectives of older people and their families whose experiences are neutral, negative, or even distressing, as well as challenge and counter existing negative stereotypes of aging in the public and literary realms. Neither research poetry nor lyric poetry is common in gerontological research; however, they offer the radical potential to offer insight into the lived realities of older people and their loved ones. Research poetry uses the direct words of older people, drawing on transcripts and found texts, and giving voice to people who otherwise would not be heard. Lyric poetry, by contrast, draws more heavily on literary techniques such as metaphor and direct address to evoke sensory and intimate experiences of aging and aged care. This paper presents 2 poems comparing and contrasting the respective processes and techniques of these different poetic forms to represent the imaginary, feared, and hoped-for futures of older people, including those in aged care.

In recent decades, entrenched societal taboos about aging in Western cultures have been increasingly dispelled as both public policy and research initiatives have increasingly focused on the questions of how to live well in old age and how to age in place. The emphasis in many of these narratives is on self-improvement, and on the locus of control an aging individual can exert to extend their life, remain healthy, and be connected to their community. This international push to reframe aging as a positive and healthy experience has produced important knowledge, led to improved perceptions of older people, and significant research and policy investment. However, it has also served to de-emphasize the more confronting aspects of old age, including experiences of frailty, physical, and cognitive impairments, and other disabilities, as well as the continuing societal reliance on care in home and institutional settings. The overriding focus on “healthy aging” to the exclusion of other narratives can also lead to a corollary perception among older people that if they have not managed to age “well” or “healthily,” they have failed (Calasanti, 2016; Cruikshank, 2013; Laceulle, 2018; Lamb, 2014).

In an Australian context, the recent Royal Commission into Aged Care Quality and Safety exposed the widespread failures of care and neglect in Australia’s home and residential aged care systems. The Royal Commission emphasized the discrimination, segregation, and isolation experienced by aged care residents, and noted that widespread ageism has led to residents’ voices and experiences not being heard and valued. As the Royal Commission observed, neglect in aged care has continued unabated, with relatively little community engagement or awareness accorded to this important social problem. Media coverage of abuse and neglect in aged care has often focused on depersonalized and anonymized accounts, leading to an absence of individual stories, faces, and voices of older people featured in the stories purportedly reporting on their experiences (Thomson et al., 2022).

Although narratives of healthy aging and aging in place have been pivotal in encouraging greater contemplation of our mortality and old age, so too is the need for a nuanced, balanced, and complete picture of what it means to grow old, including the vulnerability, frailty, and reliance on care that can also accompany our later stages of life (Higgs & Gilleard, 2015; Rubinstein & de Medeiros, 2015). Although this critical social gerontology lens offers a valuable frame for challenging dominant socio-cultural stereotypes (see Katz & Calasanti, 2015), so too does the arts and humanities. This paper explores the ways in which poetry—in both its literary and research forms—offers the capacity to represent the full physical and emotional spectrum of aging, including the perspectives of older people and their families whose experiences are neutral, negative, or even distressing in language that is direct, immediate, and emotive, although challenging the stereotypes associated with these stages of life. This paper argues that poetic interventions into the scientific and medicalized languages of aging offer new and complementary means of understanding and representing aging and aged care.

## Literary and Artistic Explorations of Aging

Literary and artistic explorations of conditions such as dementia, Alzheimer’s and Parkinson’s disease, disability and frailty, and psychological and physical states of vulnerability offer new windows into conditions that have been mostly considered from scientific, medical, and sociological standpoints. Poetry—an ancient artform that is one of the oldest forms of oral storytelling, and which uses vivid, immediate, and sensory language to convey human experience—offers a particularly powerful vehicle to convey the subjective experience of aging, including aspects that may be perceived as negative or challenging.

Poetry has been broadly posited as a conduit to empathy in readers, enhancing understanding of, and compassion for, the lives of others, including those whose experiences are unlike our own. However, the relationship between literature and empathy is complex and contested, and contingent on a range of factors that affect the overall emotional response a reader may have to a text, including individual affect and the perceived fictiveness of a literary work (Keen, 2007). Likewise, although the act of empathizing with another is often framed as an absolute moral good that can produce desirable and altruistic moral behavior, empathy comes in various forms, some of which can produce negative or even anti-social behaviors and outcomes (Bloom, 2016; Breithaupt, 2019). As Bloom notes, empathy is most often predicated on connection with specific individuals, and less likely to be felt when responding to widespread statistical concerns where individual experience cannot be ascertained (2016). However, in spite of these limitations, empathy also has the positive value of allowing one to connect with the cognitive, emotional, and bodily situation of another (Breithaupt, 2019), offering insight not into the full subjectivity of another, but rather offering a feeling of identification and likeness with the lives of others. This paper does not claim that all poems induce empathy, nor that the empathic response, when it exists, produces changes in behavior or affect toward older people; rather, it adopts an exploratory approach to the question of how both lyric and research poems may foster insight and identification with the experiences of older people, their carers and workers in the aged care environment.

In recent decades, the power of poetry has been harnessed into an arts-based research (ABR) method, research poetry, which draws on the principles of poetic composition to transform found texts—such as interview transcripts, witness testimony, or other textual sources—into short, emotive, and direct works of art. In the context of aging, a small handful of studies have applied research poetry to depict grief, the caregiving experience, and life in aged care (Gerber et al., 2022; Miller, 2021; Miller et al., 2015), providing vivid, sensory, and emotional insight into the experiences of aging and aged care.

This paper compares two discrete but complementary forms of poetry—lyric poetry and research poetry—and considers the value of each mode in exploring and representing the experience of aging and aged care. Presenting one poem authored by a lyric poet writing in a confessional mode about her father’s Parkinson’s disease, and one poem by a practitioner of research poetry who draws on found texts for her poetic material, this paper offers a discussion of the techniques, merits, and challenges of each form of poetry, and considers the value of poetic interventions into the public discourse about aging and aged care, which is often characterized by negative and stigmatizing language and terminology that devalues both residents and care workers within the aged care system (Manchha et al., 2022).

Each author has nominated a poem that epitomizes their approach to questions of composition; by placing the two forms of poetry and two poets’ methods in conversation, this paper seeks to illuminate both the differences and commonalities across lyric and research poetry and explore the key compositional techniques inherent to each mode. However, this paper does not seek to assert that one form of poetry is superior to the other; therefore, the poems presented and discussed focus on complementary but discrete subject matter, and adopt different speaking positions. As this paper explores, the different focuses and approaches of the two poems are also suggestive and indicative of the different types of subjects that suit each form of poetry; together, the two poems presented suggest the possible spectrum of approaches practitioners of both forms of poetry may adopt in writing poems about aging and aged care.

## The Lyric Poem: Address Intimacy and Presence

Lyric poetry—the first form of poetry this paper considers—belongs to the domain of creative writing, and is a form of literary writing widely practiced by contemporary poets today. At its most basic level, a lyric poem is any short non-narrative poem where a single speaker expresses thoughts or feelings. Historically, the lyric’s origins are musical, and stretch back to Ancient Greece: as Frye (1957) observes, “the Greeks spoke of lyrics as *ta mele*, usually translated as “poems to be sung.” Yet lyric poem is not a poetic form, per se: it does not have formal rules like a sonnet or villanelle, nor a desired emotional affect, like an ode or elegy. Most contemporary lyrics are written in free verse and draw on a range of poetic techniques, most commonly imagery, metaphor, and rhyme. They often focus on a single scene, and seek to capture the textures and details, the mood and atmosphere of the setting, as well as the internal thoughts and emotions of the speaker. But the lyric poem’s quintessential defining feature is its first-person speaker and their sense of address: the speaker might be speaking to themselves, addressing some other person or entity, or directly addressing the reader, but the lyric poem commonly takes the form of speech.

This lyric address, or lyric apostrophe, as it is known, invites the reader into close proximity to the poet’s emotional and psychological world. Yet the effects of this invitation are complex, as reflected in the broad body of theory exploring the relationship between a lyric poem’s speaker and the reader. This invitation that the lyric poem extends to the reader—to think of themselves as the poem’s speaker and as the intended recipient of the poem’s message, and to imagine themselves present in the scene of the poem—is the fundamental way in which the lyric poem acts as a conduit to empathy.

Neuroscientific research has shown that poetry can provoke empathic affective responses among readers such as chills and goosebumps more usually associated with music (Wassiliwizky et al., 2017); reading poetry, we experience “imaginative identification with the experiences and voices of others” (Miller et al., 2015, p. 412). Given the intimacy of its address and the empathic affective responses it provokes in the reader, the lyric poem is a useful vehicle to consider questions of aging and aged care, which are both subjects that younger people may otherwise resist imagining (Holland-Batt, 2020).

Although aging and mortality have been the subject of lyric poetry for millennia, especially through the form of the elegy—a poem that expresses grief or loss, typically of a loved one—much of this corpus of poetry has focused on grieving or protesting death and old age and viewing both states as threatening and unwelcome. Well-known examples of literature about aging and dementia include Shakespeare’s *King Lear,* W.B. Yeats’s “Sailing into Byzantium,” Dylan Thomas’s “Do Not Go Gentle into that Good Night,” or T. S. Eliot’s “Gerontion,” Philip Larkin’s “The Old Fools,” and many others yet, some of which may arguably offer negative portrayals of aging and dementia as foreign, disorienting, and disturbing states worthy of lamentation. Lyric poetry is not immune from establishing and perpetuating negative stereotypes of portrayals of ageing; like all forms of art, it has the capacity to reflect community attitudes and to exemplify the fear and resistance that can also accompany contemplation of old age.

Beyond the long-established poetic forms that contemplate aging, such as the elegy, the epitaph, and the *carpe diem* poem (Simonsen, 2019), contemporary poets demonstrate an increasing interest in subjects such as dementia and related neurological and physical decline (Zeilig, 2014), and psychological research continues to explore the therapeutic value of self-expression through poetry in aged care and other institutional environments (Swinnen, 2016). Yet lyric poems set in the environment of aged care are still relatively rare; as Simonsen (2019) observes, “literary gerontology has prioritized narrative prose fiction over versified poetry.” The lyric poem offers significant opportunities to both address the reluctance to imagine aing and aged care and to contemplate all aspects of the aging experience, including inviting empathy for states of vulnerability as well as experiences of care among older people and their families.

## The Research Poem: Working With the Voices of Others

Research poetry is an adaptation of literary poetry with the objective of communicating data through literary means. Merging “the tenets of qualitative research with the craft and rules of traditional poetry” (Leavy, 2009, p. 64), this ABR form is variously labeled research poetry, transcript poems, poetic transcription, or poetic inquiry (Faulkner, 2007; Galvin & Prendergast, 2016). A research poet takes and rearranges words from a source text, which could be traditional (e.g., interview transcripts, books, magazines, speeches, policy documents) or nontraditional (e.g., tweets, junk mail, court transcripts, product packaging), to create a poem. Similar constraints of composition are present among certain schools of literary poetry, such as the Oulipo movement, postmodern poetry, and newer related forms of “uncreative writing” (Goldsmith, 2011; Perloff, 2010) but with different motives and ends: broadly, the research poet’s objective is a distillation of the source text to generate emotional affect, whereas the literary poet drawing on similar techniques does so to call into question ideas of originality and authorship, and the limits of what poetry is and can be.

This research poem creation process involves two deceptively simple steps: (1) reading the data to search for key words, phrases, and sentences, in a process that is analogous to data reduction in qualitative analysis; and (2) arranging and rearranging participants’ words to craft a poem, drawing on established poetic techniques such as rhythm, rhyme, repetition, sound, synthesis, imagery, alliteration, metaphor, emotion, and humor. Critical to this data representation process is what Poindexter (2002) describes as “diamond-cutting”—excising words and condensing language into an impactful narrative. As a form of experiential storytelling, the best research poetry gives authentic voice to participants’ experiences and fosters empathetic understanding by “communicating instances where we feel truth has shown its face” (Richardson, 1998, p. 451).

As the research poet purposely resists dominant forms of academic discourse, not all researchers will find it within “their internal tool kits to work with data in this way” (Sparkes & Douglas, 2007, p. 186). Interested novice research poets must actively engage with the craft of poetry, learning about poetic traditions, aesthetics, and techniques as their poetic voice emerges. In encouraging research poets to consider the poetic process and the craft of poetry, Faulkner (2007) proposed six poetic criteria, what she labeled *ars poetica* (the art of poetry, see Table 1) for thinking about, reading, and evaluating research poetry: (1) artistic concentration; (2) embodied experience, (3) discovery and/or surprise, (4) conditionality, (5) narrative truth, and (6) transformation.

**Table 1.** Applying Faulkner’s (2007) Six Ars Poetica Criteria to the Research Poem

Criteria	Critical reflection of research poem
1. *Artistic concentration*—careful attention to the detail, patterns and feeling used in poetry (e.g., rhyme, titles metaphor, repetition)	This poem utilizes multiple poetic approaches (word choice, spacing, alliteration, anaphora) to engage the reader. The rhetorical technique of anaphora (where a word expression or phrase is repeated) is used in the fifth (“*to feel protected, to feel safe, to feel like they matter*”) and sixth stanza *(“help us, to help them”*), although the title—“Resident-Staff Ratio”—is specific and descriptive. Although the poem may have been strengthened by greater inclusion of metaphor and imagery, the research poet in this case has been constrained by the source text and linguistic choices of the speaker. The title is blunt and deliberately contrasts with the more emotive text of the poem in order to emphazise that these experiences the speaker recounts might be addressed with policy reform in the form of legislated staff to resident ratios.
2*. Embodied experience—*extent to which the reader feels and connects emotionally to the poetic narrative	The poem seeks to cultivate emotional connection with the reader through the experiences of a new-ish carer who is carrying the burden of too many care tasks. The speaker is extremely concerned about the residents who are waiting for care. As she notes: “*they deserve proper care, proper staff ratios, proper dignity*.” The direct second-person address demands action and invites the reader to imagine this as their own experience— “*they deserve to feel like they matter; as do we all—when it’s our time*”). The speaker’s call for this “*message, to be read, heard, out loud”* is also a subtle reminder that, for too often, the voices of residents and care staff have not been heard. The poetic narrative focusses on the immediate here and now, although asking the reader to contemplate their own future aging and vulnerability.
3. *Discovery/surprise*—extent to which reader gains a new perspective on the topic presented in the poetry.	The poem educates on multiple levels. As well as the description of all the health conditions residents have (disabilities, mental health struggles, dementia, skin conditions, fluid restrictions, and palliative care), the speaker outlines the many tasks in her work day (toilet, shower, changing clothes, retrieve items lost, preventing falls etc.). There is a strong sense that there is simply too much to do—and the surprise at the end of the poem is the strong communal call to action: “*please present change—we cannot do it alone, without you*.”
4*. Conditionality*—the point of view used to present the story	The poem presents the authentic voice and unique lived experience of an aged care worker, with only condensation of their words and no additions.
5*. Narrative truth*—the facts as presented ring true	The poem accurately reflects the subjective perspective of a personal care worker in her own words, with specific details drawn from her daily experiences in her workplace. From the specific illnesses residents experience to the time pressures the worker experiences, the poem reflects the perspective of many aged care workers that their workforce needs legislative, policy changes—as Kate explains “*we cannot do it alone, without you*.”
6*. Transform*—extent to which poem encourages new perspectives, insight, or social change	The poem has significant potential to transform, speaking directly to a potential public policy change: mandated care hours in aged care. There is also new insight, as the reader may not have been fully aware of the tasks and challenges facing the aged care workforce. Although the poem does not explain the policy reform that will be required to address the issues raised by the speaker, it does subtly draw the reader’s attention to this through its title, which invites the reader to investigate the issue further.

Faulkner’s criteria diverge radically from the criteria poets or literary scholars might use to assess the skill or effect of a poem—if indeed they would seek to apply any criteria in the first place—and have been developed to specifically address the conditions in which research poetry is written: namely, that the poems are frequently drawn from legal documents, testimony, interviews, and other accounts in which the subject is sharing truthful direct personal experience to the best of their ability. Therefore, concepts that would not generally be applied to literary poetry, such as “narrative truth,” are used as evaluative tools to prompt the research poet to consider questions of fidelity to the source text, and to ask whether the poem accurately reflects the words and experiences of the human subject and their story—to the extent that they tell one. Likewise, asking whether the poem has the capacity to transform attitudes or opinions and whether the poem fosters discovery or surprise will be conditional on the reader’s aptitude, knowledge, and many other factors which cannot be quantified. The criteria are best understood as a self-assessment tool to orient the research poet, who may not have experience in creating artistic works, to key concerns animating research poetry, and its obligations to the human subject whose words and experiences it employs.

In exploring the value of poetry to gerontology, and especially our understanding of the aged care experience, the next sections present two different poems—a lyric poem written by a literary poet, and a research poem crafted by a practitioner of research poetry—and reflect on the compositional strategies and poetic techniques used in each form of poetry.

## Lyric Poetry on Aging and Aged Care: Evoking Emotion and Inviting Imagination Identification With Older People

The following lyric poem, “The Gift” (Holland-Batt, 2022), explores the experience of aging and aged care through the lens of the poet’s experiences caring for her father although he was living in aged care with Parkinson’s Disease. Holland-Batt is a literary poet who writes predominantly in the lyric vein, and whose work bears several hallmarks of confessional poetry.

Confessional poetry—a school of writing which emerged in the late 1950s in the United States by poets such as W. D. Snodgrass, Robert Lowell, Stanley Kunitz, Anne Sexton, and Sylvia Plath—is characterized by the evocation of a confession in the poem, often of a taboo nature, combined with heightened metaphoric, rhetorical, and imagistic energy. Confessional poetry shepherded many subjects into the realm of poetry—such as mental illness, suicidal ideation, abortion, and ambivalence about motherhood—that were hitherto not thought of as the proper subjects of poetry (Holland-Batt, 2021). Holland-Batt’s poems apply aspects of confessional poetic practice to the relatively underrepresented environment of aged care, and the states of vulnerability and frailty that can accompany old age.

“The Gift,” is set in the garden of an aged care home and depicts the poet’s interactions with her father as they sit together and hold a conversation. Using a single extended metaphor exploring the idea of death as a gift, the poet conveys her father’s mental health in aged care, his emotional incontinence and pathological crying, and his ambivalent longing to be released from his condition. Written in the first person, the poem invites the reader to imagine themselves in the daughter’s position:

The Gift

In the garden, my father sits in his wheelchair

garlanded by summer hibiscus

like a saint in a seventeenth-century cartouche.

A flowering wreath buzzes around his head—

passionate red. He holds the gift of death

in his lap: small, oblong, wrapped in black.

He has been waiting seventeen years to open it

and is impatient. When I ask how he is

my father cries. His crying comes as a visitation,

the body squeezing tears from his ducts tenderly

as a nurse measuring drops of calamine

from an amber bottle, as a teen at the carwash

wringing a chamois of suds. It is a kind of miracle

to see my father weeping this freely, weeping

for what is owed him. *How are you?* I ask again

because his answer depends on an instant’s microclimate,

his moods bloom and retreat like an anemone

as the cold currents whirl around him—

crying one minute, sedate the next.

But today my father is disconsolate.

*I’m having a bad day,* he says, and tries again.

*I’m having a bad year. I’m having a bad decade*.

I hate myself for noticing his poetry—the triplet

that should not be beautiful to my ear

but is. Day, year, decade—scale of awful economy.

I want to give him his present but it is not mine

to give. We sit as if mother and son on Christmas Eve

waiting for midnight to tick over, anticipating

the moment we can open his present together—

first my father holding it up to his ear and shaking it,

then me helping him peel back the paper,

the weight of his death knocking,

and once the box is unwrapped it will be mine,

I will carry the gift of his death endlessly,

every day I will know it opening in me. (Holland-Batt, 2022)

In “The Gift,” the poet draws on a number of poetic techniques characteristic of lyric poetry, including a strong emphasis on metaphor and imagery, and a sense of a single, dramatic scene. In its first line, the poem sets up a scene and context for the poet’s utterance: the poet is sitting with her father in the garden, although he is in a wheelchair.

But after the straightforward opening line, the poem then departs from realist language into densely symbolic and metaphorical imagery. The poet describes her father as being “garlanded by summer hibiscus/like a saint in a seventeenth-century cartouche,” offering a surprising image of an aged care resident as the subject of a work of art. The poet’s father is also depicted with a “flowering wreath” around his head, which might evoke a religious halo or crown, and her use of the adjective “passionate” calls up not only the emotional realm but also Christ’s Passion. This succession of images, with the surprising combination of flowers and religious imagery, presents the poet’s father in an unconventional way: as a figure of dignity and religious grace. “He holds the gift of death/in his lap: small, oblong, wrapped in black,” the poet says, establishing the duration of her father’s illness with the line, “He has been waiting seventeen years to open it/and is impatient.” The nature of the titular gift is made clear: it is a metaphor for the release of death.

As the poem unfolds, the poet offers context for her father’s suffering, describing his emotional incontinence, which is characteristic of neurological disorders such as Parkinson’s Disease. Yet the poet opts to describe these using a series of contrasting metaphors: her father’s involuntary crying is compared to “as a nurse measuring drops of calamine/ from an amber bottle,” to “a teen at the carwash/wringing a chamois of suds,” and to “an anemone” whose “moods bloom and retreat.” This quick succession of comparisons serves to underscore how changeable and unpredictable the condition of pathological crying can be. Other than the mention of the father’s weeping being “a kind of miracle” and a “visitation,” these images depart sharply from the initial religious imagery the poem establishes, calling up both the everyday world outside the aged care home, the natural environment, and the humanity of the nurses who care for the father too.

Although the daughter is the first-person speaker of the poem, the father is also imbued with humanity and a voice in the poem; the appearance of his dialogue and voice endows him with agency and humanity to convey the complexity of his relationship with his long illness. In the small corrections within his dialogue—“*I’m having a bad day,* he says, and tries again./ *I’m having a bad year. I’m having a bad decade*”—the poet emphasizes both the father’s confusion through his dementia and also his cognition, and expresses the father’s frustration at his inability to communicate as clearly as he would like.

Together, both the daughter’s implied speech in the poem, and the father’s explicit speech, encourage the reader to imaginatively participate in the conversation. Toward its close, the poem returns to an image introduced toward its beginning: that of the “gift” the father holds on his lap. The gift is death, which is framed as a relief that both father and daughter will embrace when it comes—but one whose timing, much like a visitation or like the father’s emotions, cannot be controlled, predicted, or pre-empted. The poem’s ambivalent and ambiguous last line—“every day I will know [the gift] opening in me”—suggests that death will both be a release and a source of grief and emotional rawness for the speaker.

Alongside metaphorical language and imagery, the poet also draws on a number of additional poetic techniques to derive effects. The poet’s decision to frame the poem as a single unbroken stanza comprising lines of roughly equal length contributes to the immersive effect, and the sense of the poem being one concentrated scene. The poet also uses enjambments—the breaking of the poetic line mid-sentence—to achieve both surprise and ambiguity, as in the line “I want to give him his present but it is not mine/ to give,” which plays on the dual senses of the word “present” as both a gift, and a present tense that daughter and father do not share, before resolving it to mean the former in the following line “to give.” The poet deploys repetitional and sequential rhetoric: the repetition of the words “day,” “year” and “decade,” which appear first in the father’s dialogue and then in the speaker’s voice, emphasize the longevity of the father’s illness. The poem also establishes a rhythm through relatively even sentence length—most sentences comprising around two to four lines—before breaking this with an extended nine-line sentence at the poem’s close, which gathers a sense of momentum and urgency that contributes to the effect of the poem’s final line, which offers an almost-taboo confession that the poet herself anticipates, and will feel relief, from her father’s release from his suffering.

“The Gift” shows how lyric literary poetry can seek to consider and explore the vulnerability, ambivalence, or negative experiences of aging and aged care. It uses a range of literary techniques associated with lyric poetry, including metaphor, simile, symbol, anaphora, epistrophe, repetition, enjambment, and both the instigation, and breaking, of patterns. The first-person speaker describes the aged care resident in the environment of aged care, with a focus on imagery, emotions, ambivalence, and the relationship between the older person and their family, showing a link to their life outside the aged care environment as well as a glimpse of their life within it.

## Research Poetry on Aging and Aged Care: Evoking Emotion and Inviting Imagination Identification With Older People and Their Carers

The research poem “Resident-Staff Ratio” is also written in the first person and set in an aged care home, but with a very different structure, context, and approach to “The Gift.” It subtly invites the reader to imagine themselves in three different roles—as a worker in an aged care, as an older person living in aged care, and, unusually, as a policymaker. In part, this reflects the unusual source text for this poem: publicly available data, specifically on of the more than 10 000 submissions to the recent Royal Commission into Aged Care Quality and Safety in Australia. Although research poets typically work with interview transcripts, the poem below was crafted from a letter from a new personal care worker, calling for resident-staff ratios in aged care.

**Resident-Staff Ratio**

I’m a personal care assistant –

new to the industry,

two years – so far.

I would love this message

to be read,

heard,

acknowledged in its entirety.

Please present change -

we cannot do it alone, without you.

My facility has

ninety residents:

disabilities, dementia,

mental health struggles,

skin conditions,

fluid restrictions,

palliative care.

We are committed to helping.

Trying to do the best we can:

toilet, shower,

changing clothes,

changing linen,

preventing falls,

transferring

from bed to chair,

chair to bed,

hoists, walkers,

meal assisting,

retrieve items lost,

preventing hazards.

Waiting for our assistance

residents become incontinent -

try to get off their chairs

accidently fall

trying to toilet themselves.

Behaviours worsen -

they feel neglected.

They deserve proper care

proper dignity

to feel protected

to feel safe

to feel like they matter

as do we all - when it’s our time.

I urge you –

legalise staff ratios in aged care.

Help us

help them.

*Kate*

The language in this poem, drawn from the testimony of an aged care worker, Kate, is descriptive, engaging, and powerful, providing clear insight into what it might be like to live and work in aged care. Compared to the lyric poem, “The Gift,” however, there is no dramatic first scene, evocative metaphors, or sensory imagery to draw the reader in. That is because the research poet is constrained by the text in front of them: research poetry amplifies participant language, and as people’s individual speaking styles vary in how colorful, engaging, and evocative they are, not all source texts will work for a poetic approach (Breheny, 2012; Öhlen, 2003). In creating participant-voiced poems, most researchers follow Glesne’s (1997) approach which emphasizes honoring participant’s speaking rhythm and syntax, and only using their actual words (even if this impacts grammatical structure and flow; see Miller, 2019), with these words and pulled from anywhere in the source data.

Each stanza in “Resident-Staff Ratio” traverses a different topic. The first stanza positions the speaker’s perspective: a personal care worker—“new to the industry,” “two years—so far.” There is a conceptual openness here: a sense that if reform does not happen, this committed and caring staff member may not stay in the sector. The second stanza is a call for communal action, as we see in this phrase: “we cannot do it alone, without you*.”* This powerful first-person address engages, with the third and fourth stanzas educating by describing the lived experience of aged care and the many and varied tasks of a personal care worker, using the poetic technique of listing to convey a sense of routine and repetition in the aged care worker’s days.

Stanzas five and six describe the consequences of staff not having enough time to deliver quality care, with the language in the last line of stanza six purposefully personal. There is an implicit reminder that we all age and we all deserve quality care—“as do we all—when it’s our time.” Aged care is thus made personal. The penultimate stanza also draws on the poetic technique of anaphora (repetition at the beginning of poetic lines or phrases) to create urgency and momentum: “to feel protected/ to feel safe/ to feel like they matter.” Stanza seven returns to the main point: an explicit first-person address call to action, with the personal language (“I urge you”; “help us/ help them”) eloquently repositioning aged care policy reform to be personal (“legalise staff ratios in aged care”). The poem vibrates with the authentic, passionate, fearful voice of this aged care worker who desperately desires change and is calling for policy reform to enable her to deliver quality care. As a tool for reflecting on and illustrating the quality of this research poem, Table 1 applies Faulkner’s (2007) six ars poetica (art of poetry) criteria to “Resident-Staff Ratio.”

## Discussion

As well as comparing and contrasting the respective processes and techniques of poetry and research poetry, our aim in this paper has been to demonstrate the value of ABR approaches, such as poetry, to gerontological researchers. Although the functions and effects of poetry are complex, and include the capacity to produce ambiguity, manipulate, idealize, or romanticize experience, as well as to perpetuate negative tropes and stereotypes, poetry also has the ability to clarify, distill and magnify, showing emotionally poignant, memorable, and often hidden moments of truth that blur the distance between self and others (Faulkner, 2009; Richardson, 1998). Critically, research poetry, as Galvin and Prendergast (2016) explain, challenges the dominant scientific quantitative discourse to remove “the false mask of academic distance and expertise to reveal, poetically, human dimensions beneath” (p. 104).

ABR methods such as poetry and research poetry are not the norm within gerontological research, yet these methods hold much potential to creatively engage researchers, policymakers, and the public in a reflexive conversation about aging, old age, and aged care. Given the relentlessly positive focus of the “healthy aging” discourse and research focus in the field of gerontology at present, centering the lived experiences of older people and their family members, using metaphors and images to enable empathic imagination with older people, and returning a focus to the subjective experiences of aging and aged care are critical to offering a more balanced view of the totality of the experience of getting old.

Fortunately, a growing body of critical social gerontology, humanities, and ABR—from performing plays to collaboratively crafting poetry in aged care (Gregory, 2011; Swinnen & de Medeiros, 2018) to integrating medical humanities into the gerontology and geriatrics curriculum (O’Neill et al., 2020)—contributes to a more reflexive, nuanced and authentic dialogue about old age that challenges the dominant stereotyping socio-cultural narratives of “decline” or “age-defying” seniors, beyond the paradigms of successful and unsuccessful, healthy, comfortable or authentic aging (Cruikshank, 2013; Laceulle, 2018; Rubinstein & de Medeiros, 2015).

### The Differences Between Lyric Poetry and Research Poetry

Although lyric poetry and research poetry share certain poetic techniques, these two poetic modes have fundamentally differing aims, constraints, and objectives, as well as distinct intended audiences and measures of relative success. Although it operates within a long history and inevitably enters into a conversation with the oeuvre of poetry that has preceded it, the contemporary lyric poem is a fundamentally literary mode with no inherent formal constraints, and therefore affords the poet choice in relation to the poems’ title, lineation and stanzaic arrangement, imagery and metaphorical language, and focus.

Originality is a vexed concept in literature—as Hirshfield (1989) observes, “every writer must at some level be aware of the way in which his or her work adds to or differs or joins with everything that has gone before,” and many theorists and writers dispute whether originality is even possible (Goldsmith, 2011; Perloff, 2010)—yet the measures by which lyric poetry is judged often include perceived originality and innovation in its language use, image-making, metaphors, voice, and style. Often originality in a literary or broad artistic sense involves subverting established parameters or traditions; other times, originality involves genuine idiosyncrasy or iconoclasm, with no clear precursors (Delbanco, 2020). The lyric poem which draws on cliché, overly familiar language, or dead metaphor, will be generally considered unoriginal, and therefore a failure. This pressure to achieve originality is connected to the implied audience of a literary lyric poem: a readership that is intimately familiar with the poetic canon.

Perhaps most importantly, the lyric poem is intended to be memorable: unlike other forms of literature, including novels, nonfiction, or memoir, the lyric poem can often be heard and understood in a single sitting, and can be memorized, remembered, and repeated (Holland-Batt, 2021), which links to its origins as a form accompanied by music (Frye, 1957). This is because a successful lyric poem is not only memorable for its subject matter—grief, happiness, lost love, for example—but also for the exact expression of those ideas.

Although the lyric poem invites intimacy with the reader, and a sense of secrets being overheard (Waters, 2000), the poet is also free to invent, to conflate events, or even to lie in the service of art, even within seemingly confessional poems: this is because in reading a literary poem, as with a novel, there is a presumption of distance between the real-life author who has written it and the poem’s speaker, no matter how closely the poem seems to invite a biographical reading. The reader of the lyric poem often will flicker between occupying both the speaker’s “I” and the “you” of the addressee, entering into a complex interplay of identifications, a “being spoken through as well as speaking” (Stewart, 1995).

By contrast, the research poem—especially the found poem—has no underlying intention to be original, given that it derives all of its words from a pre-existing text. The research poet is entirely constrained by the word choice, word order, thought patterns, and speech of the subject; given that most research poems are sourced from interview transcripts, testimony, or other more quotidian forms of language, the research poet must also grapple with the inevitable redundancies, repetition, and awkward phrasings that exist in extemporaneous speech. These factors narrow the poetic possibilities considerably, given that the research poet can only work with the existing words in the order they appear in the source text—but they also set differing expectations for both the poet’s poetic expression and the effect on the reader.

Research poems derive much of their effects from the identity of the original speaker: knowing that these everyday words and phrases—even clichés—come from staff or residents in an aged care home fundamentally changes the way they are received among readers, imbuing them with greater power and poignancy than they might otherwise achieve. Unlike lyric literary poets, the research poet is not free to introduce metaphors, similes, symbols, or other elements of metaphorical language that do not exist in the source text; this can often lead the researcher to scrounge for any images or metaphors or repetition that exist within the source text to add vividness and poetic touches to their poem.

The intended purpose and audience of lyric and research poetry also differs: research poets are writing to communicate and to educate, sharing research findings—which means that any quality judgment on research poems is based on both artistic and scientific concerns. Researchers are rarely poets—and although Faulkner has lamented that she is tired of “reading and listening to lousy poetry that masquerades as research and vice versa” (2007, p. 222), there is a space for what Lahman and Richard (2013) term “good enough” research poetry, as poetic skill develops. There are very clear aesthetic differences between “The Gift” and “Resident-Staff Ratio”; although research poetry does not enable the poetic imagination present in “The Gift,” it offers researchers different benefits: the rare capacity to convey and engage with participants’ languages, perspectives and subjectivity, and to forge a creative response. As well as unquietly and emotively amplifying participants’ voices, the process also helps to enhance researcher reflexivity, meaning that even if the final poems are not published or disseminated, integrating poetry into research practice may inform and trigger broader research insights (Faulkner, 2009).

There are also similarities in the objectives and intended effects of lyric and research poetry too. Both forms of poetry aim to be memorable, seeking to achieve an emotional effect on their readers. Both forms also aim for brevity and condensation, and both forms deploy a sense of lyric address—usually from a first-person speaker—to create intimacy and a sense of address. Both forms of poetry must establish a context for their utterance—whether that is the speaker addressing a loved one, describing a scene, or focusing in on a particular behavior or memory—and offer clarity about who is speaking to whom, and about what. And both forms of poetry—through their choices in language—seek to introduce a sense of personality in the speaker.

## Conclusion

The special language of poetry “makes the world visible in new and different ways, in ways ordinary social science writing does not allow” (Denzin, 2014, p. 86); this unique scholarship provokes “connections, open[s] spaces for dialogues, [and] heals” (Pelias, 2004, p. 2). In the context of gerontology, therefore, both lyric poetry and research poetry offer significant potential to engage with the challenging and taboo aspects of aging and aged care and broaden the discourse of aging into the imaginative, subjective, and affective realms. Like all artforms, poetry has limitations: it has the capacity to perpetuate negative stereotypes of aging and aged care as well as to critique or challenge them, and its inherent ambiguities and status as a literary artifact open it up to a broad range of interpretative communities, many of whom may disagree about its functions, significance, and value. Yet even the interpretive disagreements poetry may produce can be of benefit to the field of gerontology: poetry invites close scrutiny of language and questions of representation and invites productive discourse and disagreement. As the field continues to address the challenge of aging healthily into the future, it is critical that it also fosters new and effective ways to acknowledge the challenges, taboos, and emotional terrain that comes with aging for older people, their carers and families, and these two forms of poetic practice offer a way.

## Data Availability

This article does not report data and therefore the pre-registration and data availability requirements are not applicable.
